# Leptin-Notch signaling axis is involved in pancreatic cancer progression

**DOI:** 10.18632/oncotarget.13946

**Published:** 2016-12-15

**Authors:** Adriana Harbuzariu, Antonio Rampoldi, Danielle S Daley-Brown, Pierre Candelaria, Tia L Harmon, Crystal C Lipsey, Derrick J Beech, Alexander Quarshie, Gabriela Oprea Ilies, Ruben R Gonzalez-Perez

**Affiliations:** ^1^ Department of Microbiology, Biochemistry and Immunology, Morehouse School of Medicine, Atlanta, GA, 30310 USA; ^2^ Department of Surgery, Morehouse School of Medicine, Atlanta, GA, 30310 USA; ^3^ Biomedical Informatics Program and Master of Science in Clinical Research Program, Clinical Research Center, Morehouse School of Medicine, Atlanta, GA 30310, USA; ^4^ Department of Pathology and Laboratory Medicine, Emory University School of Medicine, Grady Memorial Hospital, Atlanta, GA, 30303 USA

**Keywords:** leptin, Notch, pancreatic cancer, pancreatic cancer stem cells, leptin peptide receptor antagonist LPrA2

## Abstract

Pancreatic cancer (PC) shows a high death rate. PC incidence and prognosis are affected by obesity, a pandemic characterized by high levels of leptin. Notch is upregulated by leptin in breast cancer. Thus, leptin and Notch crosstalk could influence PC progression. Here we investigated in PC cell lines (BxPC-3, MiaPaCa-2, Panc-1, AsPC-1), derived tumorspheres and xenografts whether a functional leptin-Notch axis affects PC progression and expansion of pancreatic cancer stem cells (PCSC). PC cells and tumorspheres were treated with leptin and inhibitors of Notch (gamma-secretase inhibitor, DAPT) and leptin (iron oxide nanoparticle-leptin peptide receptor antagonist 2, IONP-LPrA2). Leptin treatment increased cell cycle progression and proliferation, and the expression of Notch receptors, ligands and targeted molecules (Notch1-4, DLL4, JAG1, Survivin and Hey2), PCSC markers (CD24/CD44/ESA, ALDH, CD133, Oct-4), ABCB1 protein, as well as tumorsphere formation. Leptin-induced effects on PC and tumorspheres were decreased by IONP-LPrA2 and DAPT. PC cells secreted leptin and expressed the leptin receptor, OB-R, which indicates a leptin autocrine/paracrine signaling loop could also affect tumor progression. IONP-LPrA2 treatment delayed the onset of MiaPaCa-2 xenografts, and decreased tumor growth and the expression of proliferation and PCSC markers. Present data suggest that leptin-Notch axis is involved in PC. PC has no targeted therapy and is mainly treated with chemotherapy, whose efficiency could be decreased by leptin and Notch activities. Thus, the leptin-Notch axis could be a novel therapeutic target, particularly for obese PC patients.

## INTRODUCTION

Pancreatic cancer (PC) represents only 3% of the new cancer cases diagnosed in USA, but it has been the fourth cancer-related cause of death, with a five year survival rate of less than 5% for the last decade [1]. The American Cancer Society estimates that about 50,070 people (27,760 men and 25,400 women) will be diagnosed with PC, while about 41,780 people (21,450 men and 20,330 women) will die of PC in United States in 2016. PC's dismal prognosis is mostly due to the lack of early detection, the aggressive behavior of the tumors and the lack of responses to chemotherapy [2]. A variety of risk factors have been linked to PC, including smoking, diabetes, history of chronic pancreatitis and infection with H. pylori [3]. ABO blood types and genetic variants may also influence pancreatic cancer risk [4].

Obesity (BMI > 30) is pandemic in the US and has been associated with poor prognosis of several malignancies, including prostate, colon and breast cancer [5]. In addition, overweight is associated with shortened survival in patients with advanced PC, independently of known prognostic factors, including high CA19-9 serum concentration, disease stage [6], and increased lymph node metastasis in patients with resected PC [7]. Obesity was also recently shown to promote stromal desmoplasia that was linked to PC growth [8]. However, the mechanisms involved in the association between elevated BMI and decreased PC survival are still poorly understood.

A factor that could be involved in these relationships is the adipokine leptin. Leptin is a small protein (16 kD) produced mainly by adipocytes that is involved in the control of food intake, energy expenditure, reproductive function, and body weight. Leptin binding to its receptor, OB-R expressed by cells in the ventromedial hypothalamus, regulates food intake and energy metabolism [9]. Obese and overweight individuals show high levels of leptin, but frequently exhibit leptin resistance, where the adipokine cannot regulate appetite and energy balance. High levels of leptin have been associated with increased incidence, development and poor prognosis of several cancer types [10]. Recent studies showed that PC cells express OB-R [11, 12]. Moreover, a positive correlation between increased leptin levels and PC has been reported [13]. However, there are few reports on the role of leptin in PC.

Notch signaling pathway plays a critical role in cell proliferation, survival, apoptosis and differentiation, affecting the development and function of different organs [14]. Notch is a hallmark of breast and other cancer types [15]. Moreover, Notch signaling has been associated with increased PC growth and chemoresistance [16, 17]. In addition, Notch activation has been shown to prevent pancreatic epithelial differentiation, which results in increased maintenance of PCSC and drug resistance [17]. However, contradictory roles either as oncogenic or tumor suppressor factor for Notch signaling in pancreatic intraepithelial neoplasia (PanIN) and PC are still a matter of discussion. Nevertheless, several drugs targeting the activation of Notch (*i.e*., gamma secretase inhibitors) have been proposed for the treatment of PC [16].

We have previously shown that leptin induces the expression of Notch receptors and ligands in breast cancer [18]. Moreover, the inhibition of leptin signaling reduced Notch expression and development of breast cancer, which was more evident in obesity conditions [19, 20]. Here, we investigated whether leptin increases PC cell proliferation and Notch expression, and whether a leptin-Notch axis induces PCSC and tumor development in a mouse xenograft model. Present data suggest that leptin is a proliferator factor and inducer of PC tumorigenesis and PCSC. PC cells express leptin and OB-R, which suggest an autocrine/paracrine signaling loop could occur in PC. Moreover, for the first time, it was found that leptin upregulates Notch in PC, and the specific inhibition of leptin receptor delays tumor onset, decreases tumor growth and PCSC populations. These results open the possibility to target leptin signaling and/or leptin-Notch axis as a new strategy to treat PC, which is a deadly cancer influenced by obesity, with few therapeutic options and poor outcomes.

## RESULTS

### Leptin induces proliferation of PC cells

**Figure 1:**
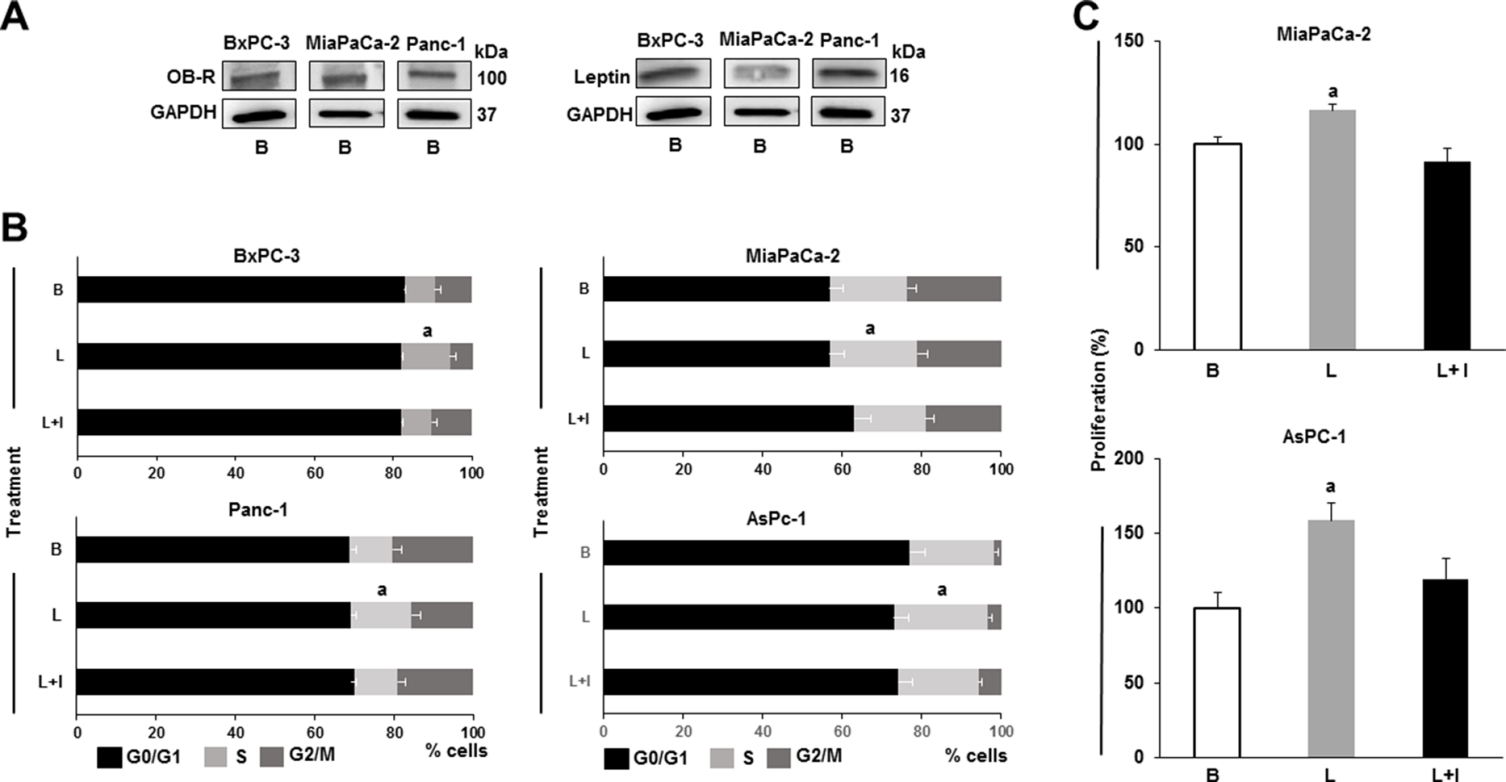
Leptin is a proliferator factor for PC cells (**A**) Leptin and leptin receptor (OB-R) are expressed in PC cells. Representative western blot results for leptin and OB-R expression. (**B**) Leptin induces PC cell S-phase progression. (**C**) Leptin induces PC cell proliferation. Whole cell lysates were analyzed by western blot. GAPDH was used as protein loading control. Cell cycle progression was measured by image cytometry. Cell proliferation was assessed using Vybrant MTT assay kit. PC cells (B×PC-3, MiaPaCa-2, Panc-1 and AsPC-1) were cultured in medium containing leptin and IONP-LPrA2. Basal condition (untreated) was used as control (100%). Effects of treatments on cell cycle progression and proliferation were expressed as % of control. Data are representative from three independent experiments; a: *p* ≤ 0.05 compared to basal condition. B = basal; L = leptin (1.2 nM); L+I = leptin (1.2 nM) + IONP-LPrA2 (0.0036 pM).

All human PC cell lines (BxPC-3, MiaPaCa-2 and Panc-1) investigated express leptin and its receptor (OB-R) (Figure 1A). To investigate whether leptin increases proliferation, PC cell lines were cultured, after starvation for 24 hours, in medium containing human recombinant leptin (1.2 nM, which is equivalent to leptin serum levels in overweight patients). Leptin significantly increased PC cell cycle progression (Figure 1B). The number of Panc-1, MiaPaCa-2 and AsPc-1 cells in the S phase was augmented by leptin. However, BxPC-3 cells were less responsive to leptin-induced proliferation (Figure 1A). Results from MTT assay show that leptin significantly induced proliferation of MiaPaCa-2 and AsPC-1 cells. The addition of leptin signaling inhibitor (IONP-LPrA2) suppressed leptin-induced growth of PC cells.

### Leptin induces Notch expression in PC cells

**Figure 2:**
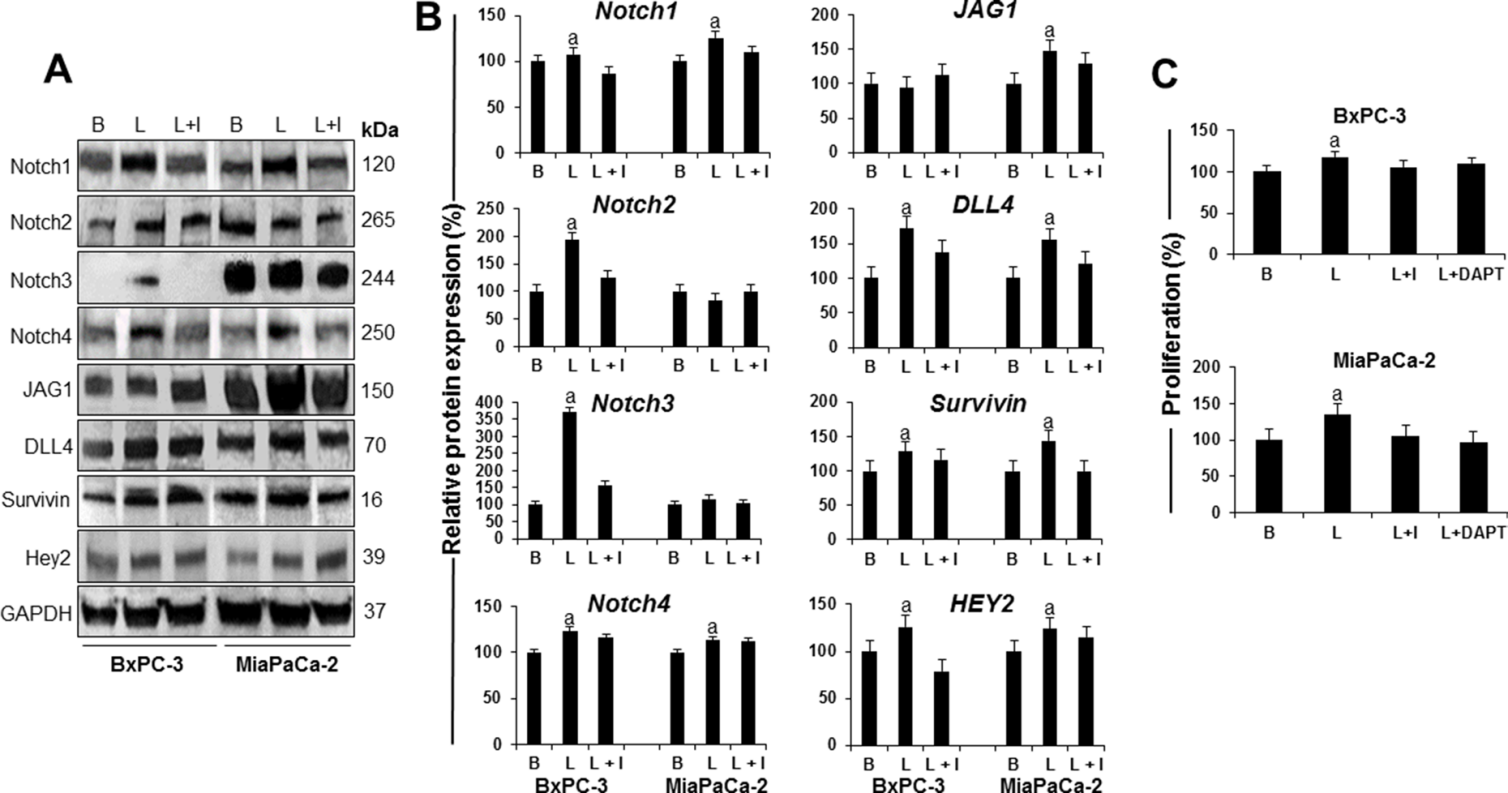
Leptin-induced Notch is linked to proliferation of PC cells (**A**) Representative western blot results of leptin induction of Notch receptors, ligands and targeted molecules in PC cells. (**B**) Quantitative analysis of leptin-induced effects on Notch, ligands and targets. (**C**) Effects of DAPT (a gamma secretase inhibitor) and IONP-LPrA2 (a leptin signaling inhibitor) on leptin-induced proliferation of PC cells. BxPC-3 and MiaPaCa-2 cells were treated with leptin and IONP-LPrA2 for 24 hours. Whole cell lysates were analyzed by western blot. GAPDH was used as protein loading control. Proliferation was determined by MTT assay. PC cells were treated with leptin, IONP-LPrA2 and DAPT for 24 h. Basal (untreated) condition was used as control (100%). Data are expressed as % of control and represent at least three independent experiments. B = basal; L = leptin (1.2 nM); I = IONP-LPrA2 (0.0036 pM); L+I = leptin (1.2 nM) + IONP-LPrA2 (0.0036 pM); L+DAPT = leptin(1.2 nM) + DAPT (20 μM); a: *p* ≤ 0.05 compared to basal condition.

To determine whether leptin regulates Notch, BxPC-3 and MiaPaCa-2 cells were treated with leptin. Western blot (WB) results show that leptin induces Notch expression in PC cells (Figure 2A and 2B). Leptin significantly increased Notch1, Notch2, Notch3 and Notch4 receptors as well as DLL4 ligand in BxPC-3 cells, and Notch1 and Notch4 receptors, and JAG1 and DLL4 ligands in MiaPaCa-2 cells. Additionally, leptin significantly increased the levels of Notch targeted molecules, survivin and Hey2, in both BxPC-3 and MiaPaCa-2 cells. IONP-LPrA2 decreased leptin's effects that assessed the specificity of leptin-induced changes on the levels of Notch components. Next, it was investigated if leptin-induced PC proliferation involves Notch signaling. The abrogation of Notch signaling by DAPT, a gamma-secretase inhibitor, decreased leptin-induced proliferation of BxPC-3 and MiaPaCa-2 cells (Figure 2C). These results indicate that Notch is required for leptin-induced PC proliferation.

### Leptin increases PC stem cell (PCSC) populations

**Figure 3:**
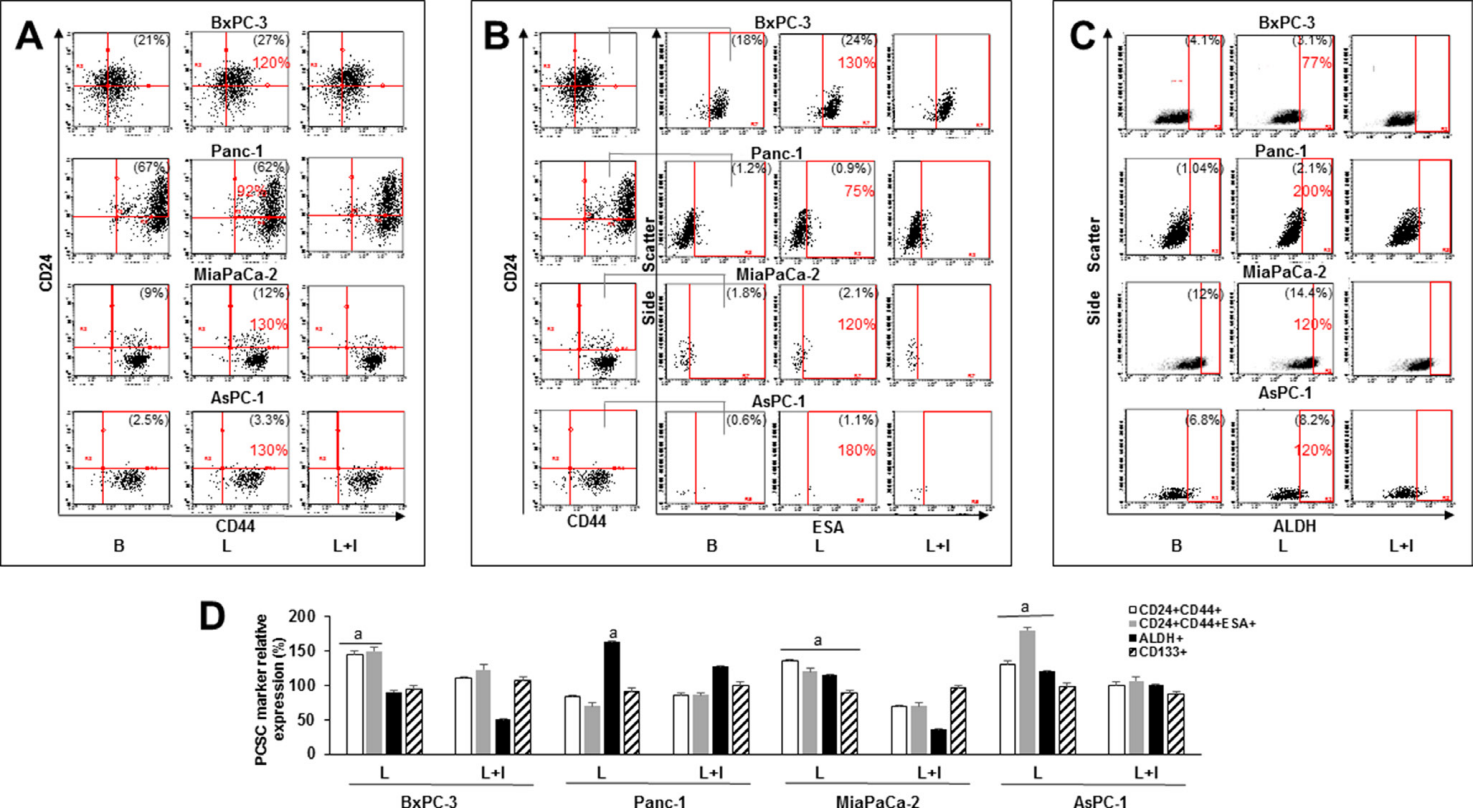
Leptin increases PC stem cell (PCSC) populations Representative dot plots of PC cells expressing (**A**) CD24+CD44+, (**B**) CD24+CD44+ESA+ and (**C**) ALDH+. (**D**) Relative expression of CD24+CD44+, CD24+CD44+ESA+, ALDH+ and CD133+ in PC cell lines. PC cells were cultured in medium containing leptin and IONP-LPrA2 for 24 hours (AsPC-1), 48 hours (Panc-1) and 72 hours (BxPC-3 and MiaPaCa-2). The expression of PCSC markers was determined by flow cytometry analysis. Basal condition (untreated) was used as control (100%). Effects of treatments on PCSC were expressed as % of control. Percent of positive cells for the PCSC markers is shown in parenthesis in basal and leptin-treated conditions. Percent of change compared to basal is shown in red. All experiments were performed in triplicate. B = basal; L = leptin (1.2 nM); L+I = leptin (1.2 nM) + IONP-LPrA2 (0.0036 pM IONP); a: *p* ≤ 0.05 compared to control.

To further investigate the potential contribution of leptin to the development of PC, the cells were cultured with leptin in culture plates to form monolayers or in low-attachment plates to allow floating tumorsphere formation. In PC monolayers, leptin increased the expression of PCSC cell surface and functional markers CD24 and CD44 in BxPC-3, MiaPaCa-2 and AsPC-1 cells (Figure 3A). Moreover, leptin increased the number of BxPC-3, Mia-PaCa-2 and AsPC-1 cells co-expressing CD24, CD44 and ESA (Figure 3B). PCSC markers (CD24, CD44, ESA) were increased higher by leptin in the metastatic cell line AsPC-1 (Figure 3D). In addition, ALDH activity was increased by leptin in the more aggressive PC cells (Panc-1, MiaPaCa-2 and AsPC-1) (Figure 3C). However, leptin did not increase CD133+ expression in PC cells (Figure 3D). These data underscore the heterogeneity of PC cells, and suggest that leptin-induced effects on PCSC are cell line dependent. Furthermore, the inhibition of leptin signaling using IONP-LPrA2 reduced leptin's effects.

**Figure 4:**
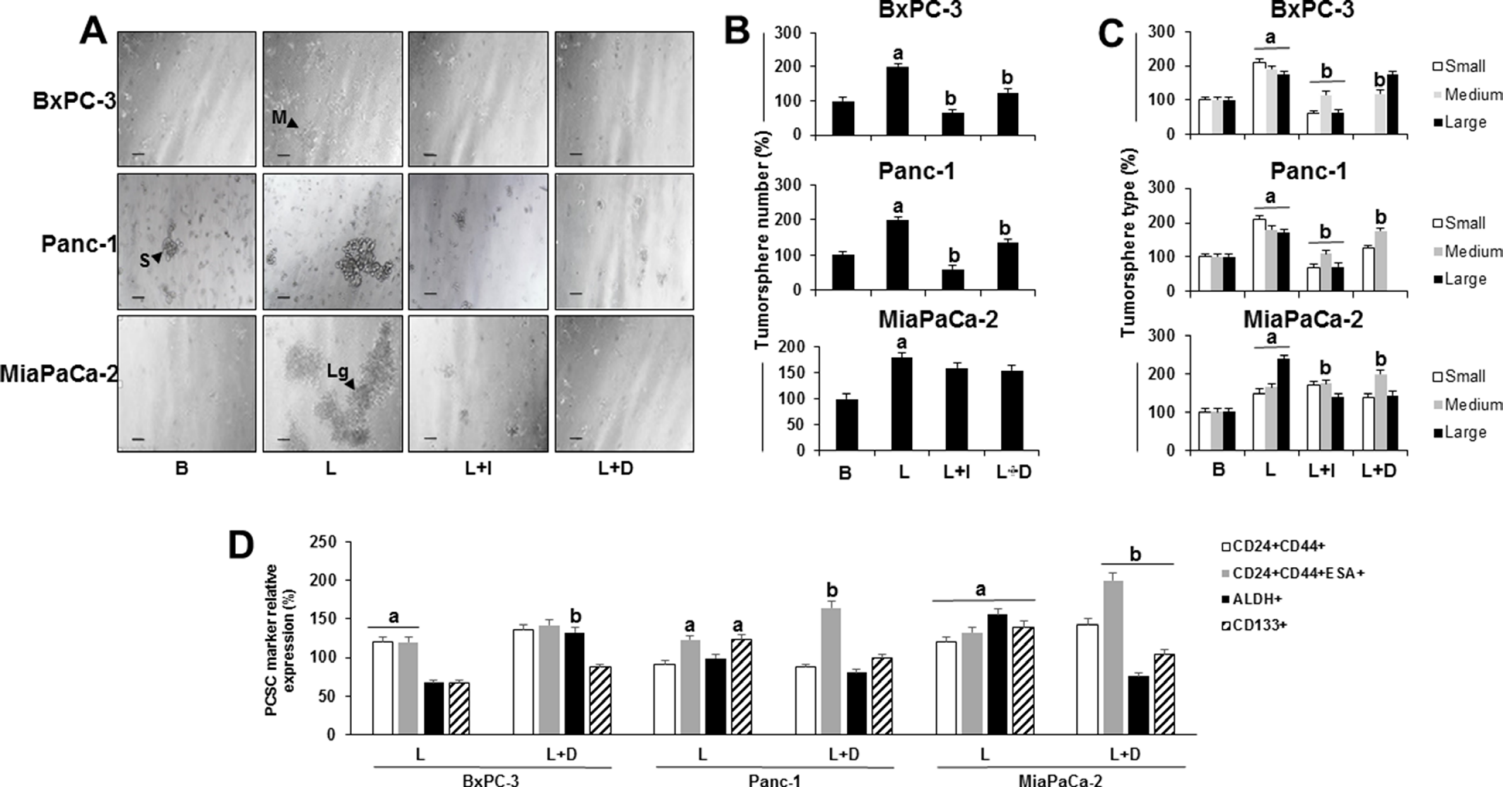
Effects of leptin and Notch on number and size of primary tumorspheres and PC stem cells (PCSC) (**A**) Representative images of tumorspheres (scale bar = 60 μm) (**B**) Number of PC tumorspheres (**C**) Number of PC tumorspheres by size (**D**) Relative expression of PCSC markers in cells from PC tumorspheres. BxPC-3, Panc-1 and MiaPaCa-2 cells (20,000 cells/well in low attachment plates) were cultured in Mammocult medium containing leptin, IONP-LPrA2 and DAPT (γ-secretase inhibitor-GSI) for 1 week. Tumorsphere number and size were determined under microscope and PCSC markers were assessed by flow cytometry analysis. Basal condition (untreated) was used as control (100%). Effects of treatment on tumorspheres and PCSC markers was expressed as % of control. All experiments were performed in triplicate. a:*p* ≤ 0.05 when compared to control; b: *p* ≤ 0.05 when compared to leptin. B = basal; L = leptin (1.2 nM); L+I = leptin (1.2 nM) + IONP-LPrA2 (0.0036 pM); L+D = leptin (1.2 nM) + DAPT (20 μM); S = small tumorsphere; M = medium tumorsphere; Lg = large tumorsphere.

Leptin also increased the number and size of primary PC tumorspheres (Figure 4A–4C). Furthermore, leptin had similar effects on number and size of secondary (P2) and tertiary (P3) BxPC-3 and Panc-1 tumorspheres. Also, PCSC (CD24+CD44+ and CD24+CD44+ESA+) were increased by leptin in P2 and P3 tumorspheres (see Supplementary Figure S1). Leptin was detected in the conditioned media from untreated (basal) P2 tumorspheres (Supplementary Figure S1). IONP-LPrA2 decreased leptin's effects on tumorsphere size (Figure 4C). Additionally, DAPT reduced leptin-induced increase of tumorsphere number in BxPC-3 and Panc-1 cells. Moreover, BxPC-3 cells treated with DAPT showed no formation of small tumorspheres. Similarly, Panc-1 cells treated with DAPT showed no formation of large tumorspheres. In MiaPaCa-2 cells, DAPT also reduced the formation of large tumorspheres.

Leptin increased PCSC markers CD24+CD44+ESA in all tumorspheres (Figure 4D), ALDH in MiaPaCa-2, and CD133+ in Panc-1 and MiaPaCa-2 tumorspheres (Figure 4D). The inhibition of Notch signaling by DAPT abrogated leptin-induced changes in tumorspheres (Figure 4A–4C), but did not affect leptin-induction of CD24+CD44+ (Figure 4D). PC cells treated with leptin and DAPT showed a further induction of cells expressing CD24+CD44+ESA+. In contrast, the inhibition of Notch signaling reduced leptin induction of CD133+ and ALDH+ cells (Figure 4D).

In basal condition, higher number of CD24+CD44+ cells were found in tumorspheres (BxPC-3 and MiaPaCa-2) compared to cells growing in monolayers. However, CD24+CD44+ESA+ cells were increased in Panc-1 and BxPC-3 tumorspheres. In addition, both ALDH+ and CD133+ cell populations were increased in tumorspheres compared to monolayers in all cell lines studied (Supplementary Table S1).

### Leptin stimulates the onset and growth of PC xenografts, and the expression of PCSC markers

**Figure 5:**
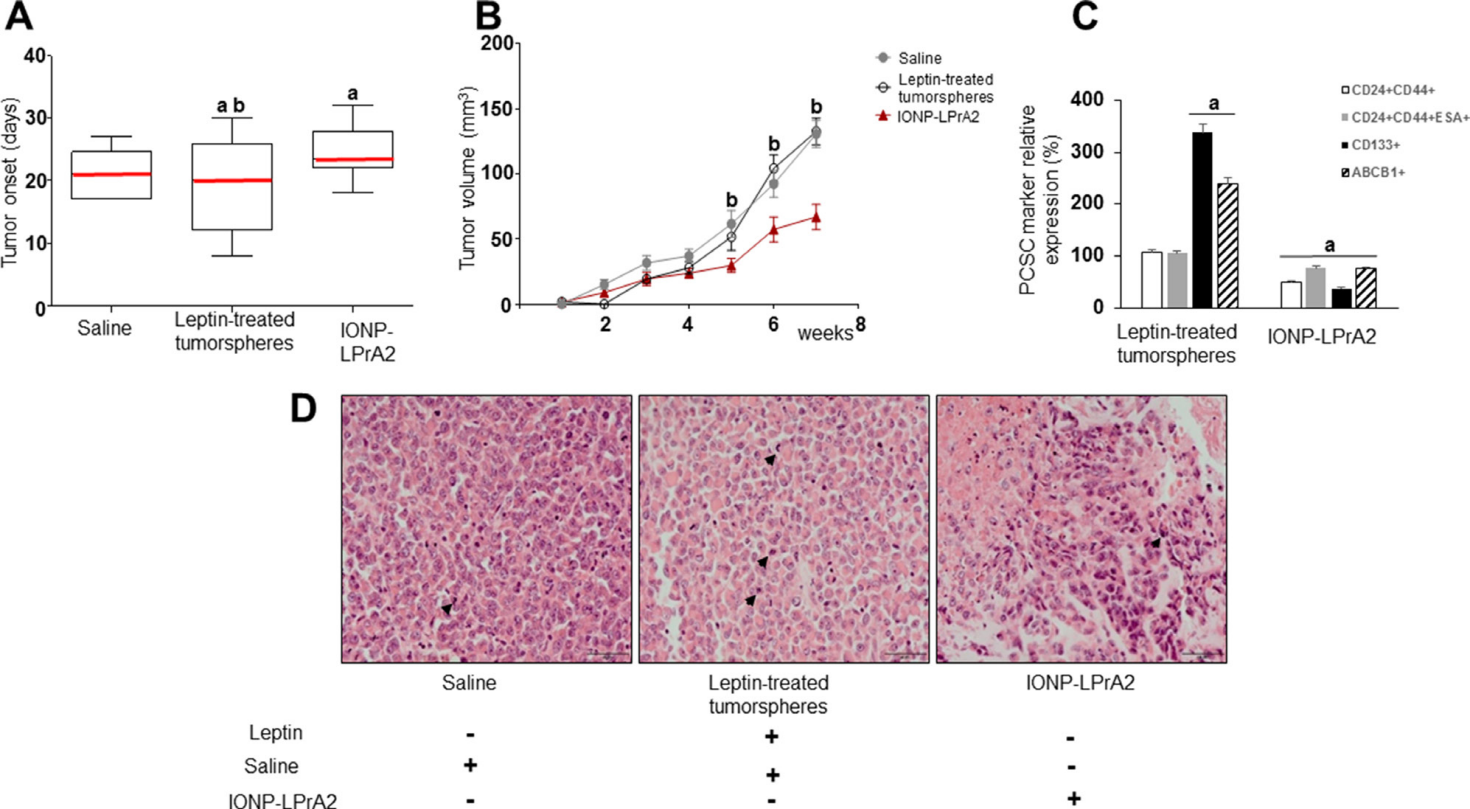
Leptin induces PC xenograft onset, growth and cancer stem cells (PCSC) (**A**) PC xenograft onset (**B**) PC xenograft growth (**C**) Relative expression of PCSC markers in PC xenografts (**D**) PC xenograft histology. H&E stained tumor sections after 7 weeks of treatment. One representative tumor specimen is presented; black arrow heads point to PC cells in mitosis. Immunocompromised mice (*n* = 21) were implanted in their flanks with MiaPaCa-2 cells (5,000 cells/matrigel 1:1) obtained from tumorspheres cultured in basal medium (*n* = 14) or containing leptin (1.2 nM; *n* = 7). The mice implanted with PC cells cultured in basal medium were treated with saline (sham control; *n* = 7) or IONP-LPrA2 (50 μl/0.0036 pM; *n* = 7) twice per week for 7 weeks. Mice implanted with cells from leptin-treated tumorspheres received saline (*n* = 7). Mean values of tumor onset in the treatment groups are represented by a red line. PCSC markers were determined by flow cytometry analysis. Mice treated with saline were used as control (100%). Effects of treatment on PCSC markers were expressed as % of control. All experiments were performed in triplicate. a:*p* ≤ 0.05 compared to saline; b:*p* ≤ 0.05 compared to IONP-LPrA2.

To investigate whether leptin signaling could impact on PC development, a heterotopic PC xenograft mouse model was used. Tumorspheres formed from MiaPaCa-2 cells were either untreated or treated with leptin for ten days, and implanted subcutaneously in nude mice. Mice implanted with untreated tumorspheres received treatment with saline or IONP-LPrA2 intravenously twice a week. All mice implanted with PC tumorspheres showed tumor development. However, early tumor onset was detected in mice implanted with leptin-treated tumorspheres (Figure 5A). Mice treated with IONP-LPrA2 showed delayed tumor onset (Figure 5A), and decreased tumor growth (Figure 5B).

Histologic analysis of PC xenografts derived from MiaPaCa-2 cells showed high-grade tumors. Tumors showed large cells, with fair amount of cytoplasm, high nuclear-to-cytoplasm ratio and nuclei frequently eccentrically located. In addition, the nuclei were either hyperchromatic or showed chromatic clearing and prominent, cherry red nucleoli (Figure 5C). Remarkably, PC from leptin-treated tumorspheres showed significantly less necrosis and frequent mitosis (up to 61 cells in mitosis/10 HPF with a maximum of 11 cells in mitosis/ 1HPF) (Supplementary Table S2). Cells from PC xenografts were further investigated for the expression of PCSC markers via flow cytometry. Leptin-treated tumorspheres led to PC xenografts with increased expression of CD133 and ABCB1 proteins compared to saline treatment (Figure 5D). IONP-LPrA2 treatment significantly reduced PCSC markers within PC xenografts (Figure 5D). These results indicate that leptin signaling induces early onset, tumor growth and expression of PCSC markers in PC xenografts. Moreover, leptin increased ABCB1+ cells, suggesting that this adipokine could be involved in the development of PC chemoresistance. Notably, IONP-LPrA2 treatment reduced PC growth, but did not induce changes in mouse body weight, food intake or general health.

### Inhibition of leptin signaling reduces the expression of factors involved in PC progression

**Figure 6:**
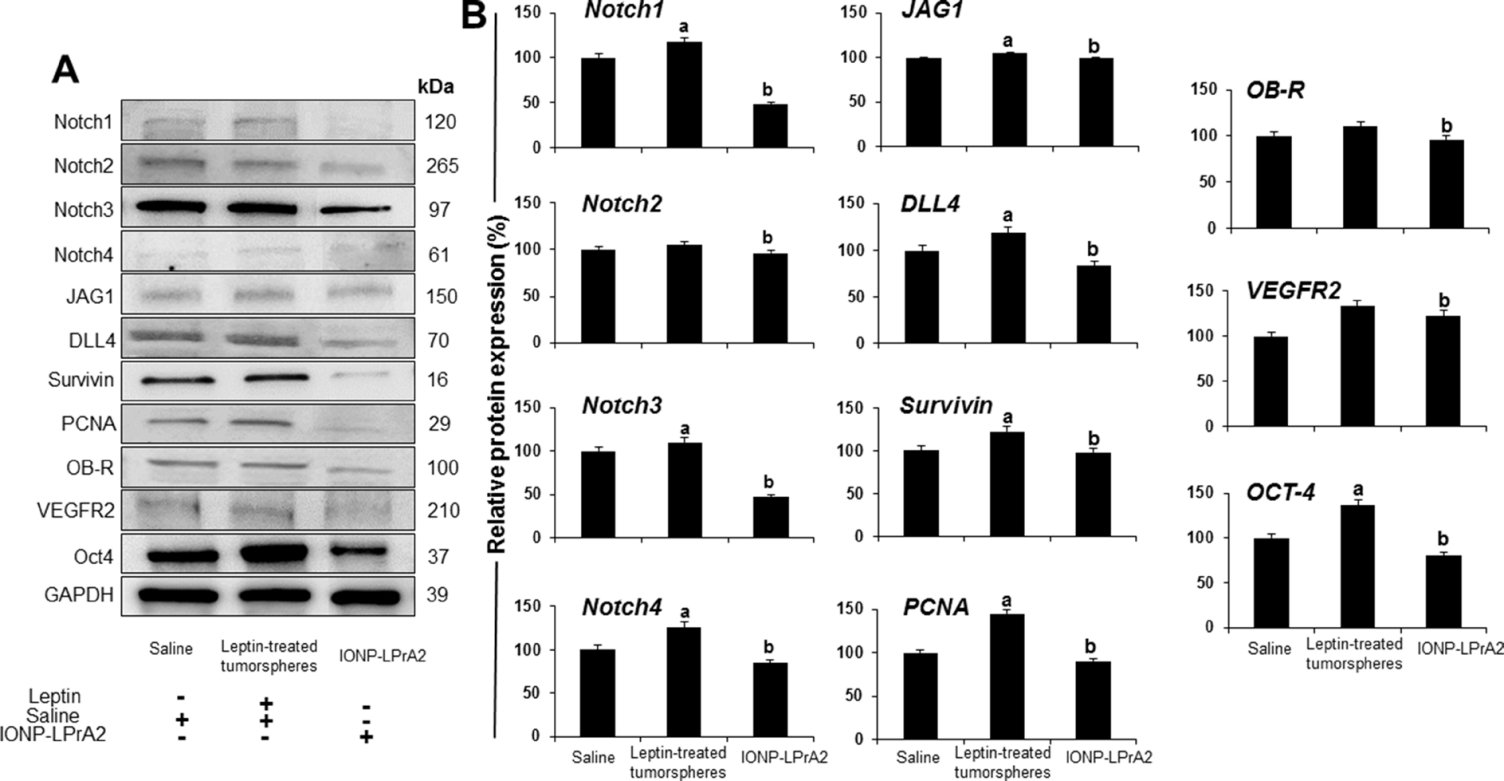
Inhibition of leptin signaling reduces the expression of Notch and related molecules in PC xenografts (**A**) Representative western blot results of PC xenografts. (**B**) Quantitative determination of tumor proteins. Immunocompromised mice (*n* = 21) were implanted in their flanks with MiaPaCa-2 cells (5,000 cells/matrigel 1:1) obtained from tumorspheres cultured in basal medium (*n* = 14) or containing leptin (1.2 nM; *n* = 7). The mice implanted with PC tumorspheres cultured in basal medium were treated with saline (sham control; *n* = 7) or IONP-LPrA2 (50 μl/0.0036 pM; *n* = 7) twice per week for 7 weeks. Mice implanted with cells from leptin-treated tumorspheres received saline (*n* = 7). Tumors were harvested and used to obtain protein lysates that were analyzed by western blot. GAPDH was used as protein loading control. Mice treated with saline were used as control (100%). Effects of treatment on the expression of Notch and related molecules were determined as % of control. a: *p* ≤ 0.05 compared to saline; b: *p* ≤ 0.05 compared to leptin.

To gain a better understanding of the role of leptin in PC, tumors were lysed to investigate protein expression. WB analyses of pooled tumor lysates showed that PC xenografts derived from leptin treated-tumorspheres had significantly higher levels of Notch4, DLL4, JAG1, survivin, PCNA and Oct-4. Remarkably, PC xenografts from mice treated with IONP-LPrA2 showed reduced expression of Notch (receptors and ligands), as well as Notch targeted molecules: survivin, PCNA, VEGFR-2 and Oct-4. Additionally, IONP-LPrA2 treatment reduced the levels of leptin receptor, OB-R, in tumors (Figure 6A and 6B). These results strongly suggest that leptin is a proliferator factor for PC, which may act, at least partially, through the induction of Notch pathway.

## DISCUSSION

Present data assess that PC cells express OB-R, and show for the first time that leptin is secreted by PC cells and tumorspheres enriched in PCSC. Further, we also demonstrated for the first time that leptin can induce Notch and PCSC in several PC lines, derived tumorspheres and xenografts. Moreover, these leptin's effects were associated with significant increase of tumorigenic features, proliferation, cell cycle progression, PCSC markers, and expression of an ATP-binding cassette transporter protein, ABCB1.

**Figure 7:**
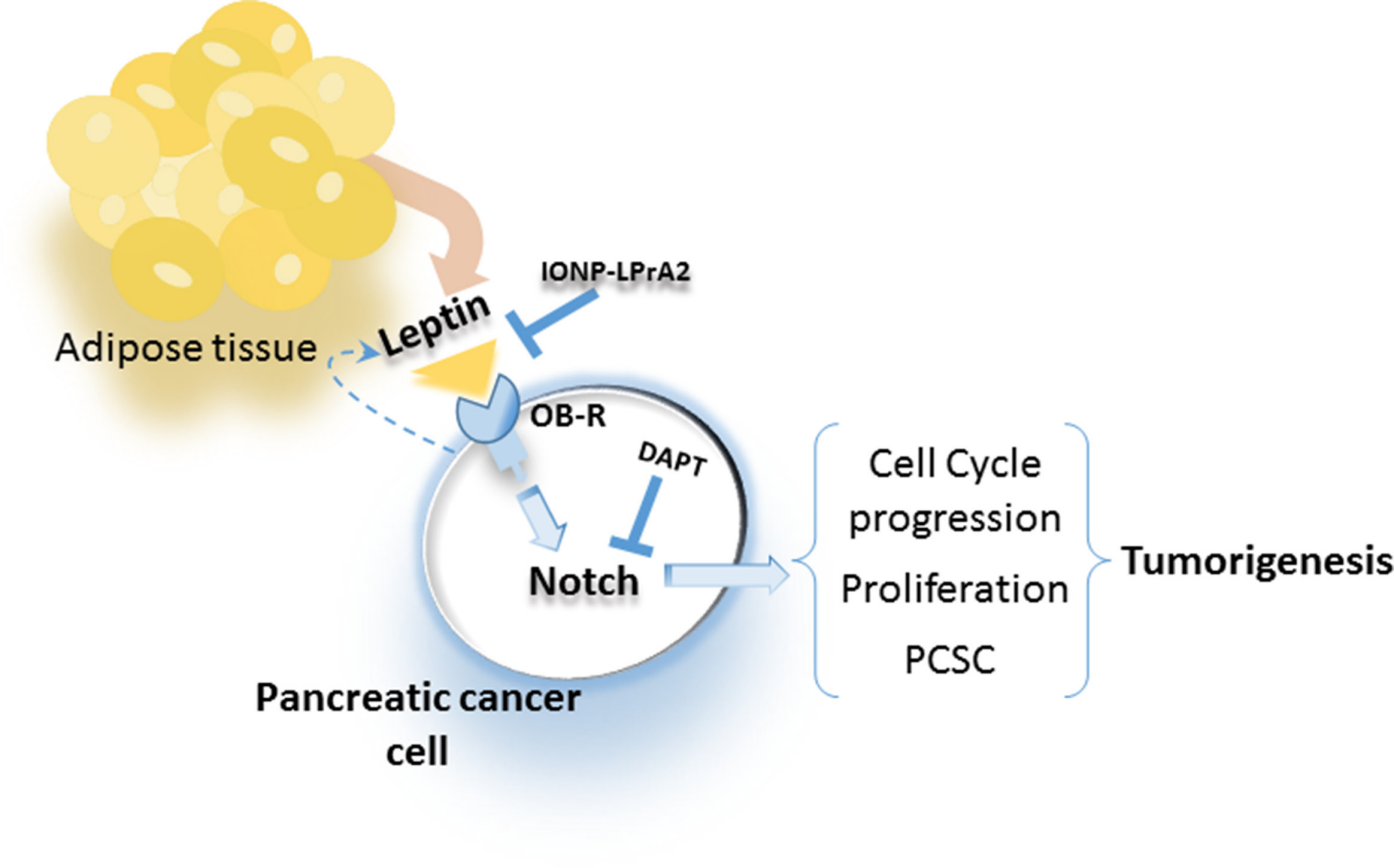
Leptin secreted from adipose tissue and PC cells binds to OB-R that increases Notch expression, cell cycle progression, proliferation and PCSC, which increase tumorigenesis Blockade of leptin signaling via IONP-LPrA2 or inhibition of Notch via gamma-secretase inhibitor (DAPT) reduces leptin actions on PC cells.

Remarkably, the blockade of Notch activation via inhibition of gamma secretase significantly reduced leptin's effects on PC cells and tumorsphere formation. Moreover, the inhibition of leptin signaling *in vivo* using IONP-LPrA2 delayed the onset and decreased the growth of PC xenografts that was concurrent to the decrease of Notch, and PCSC and proliferator markers in tumors. Taken together these data suggest that a functional leptin-Notch axis takes place in PC cells, which could induce tumorigenesis, metastasis and drug resistance due to leptin-induced expansion of PCSC populations (Figure 7).

Obesity is associated with increased PC risk, advanced stage at diagnosis and poor survival [21]. Moreover, obesity influences the development of fatty pancreas, and potentiates the growth of PC [13]. It has been suggested that these obesity's effects could be linked to leptin signaling, which is the main adipokine secreted by adipocytes, and is also synthesized by cancer cells. Leptin levels are higher in obese individuals, which has been previously linked to the progression of several cancers [10]. This adipokine is a proliferator, pro-angiogenic, anti-apoptotic and inflammatory factor that can affect both tumor and stromal cells [15, 18, 22, 23].

We have previously found that leptin induces the growth of mammary tumors in syngeneic [19, 24], and immunocompromised mice [25]. Leptin also promotes the development of carcinogenic-induced tumors in mice [20]. Likewise, leptin has previously been linked to PC growth [13]. Present data further assessed that leptin is a growth factor for PC cells. Leptin at concentrations comparable to those found in overweight patients (1.2 nM equivalent to 20 ng/ml) induced proliferation of all PC cell lines tested, formation of primary, secondary and tertiary tumorspheres and increased PCSC populations, which are features linked to the enhancement of PC tumorigenesis potential. Moreover, inhibition of leptin signaling via IONP-LPrA2 delayed tumor onset, reduced PC xenograft growth, which correlated to leptin-induced Notch expression and PCSC.

Although the molecular causes of PC are largely elusive, studies have demonstrated that multiple critical genes, including K-Ras, p53, p16, and other key cellular signaling pathways such as PI3K/Akt, mammalian target of rapamycin (mTOR), nuclear factor-kappa B (NF-κB), epidermal growth factor receptor (EGFR), and sonic hedgehog (SHH) play important roles in the pancreatic tumorigenesis [26].

Obese mice developed larger tumors, showed significantly greater number of metastases and mortality than lean mice [27]. These observations were further assessed in diet induced obesity (DIO) mice hosting orthotopic syngeneic PC xenografts [12]. DIO-mice showed increased body and pancreatic weights, and size of peripancreatic adipocytes. These changes were associated with leptin/OB-R induced activation of the PI3K/AKT pathway, which also promoted tumor cell migration. Moreover, siRNA OB-R knockdown abrogated DIO-associated PC growth [12]. Elastase (EL)-Kras mice showed increased earlier onset, increased frequency and growth of neoplastic lesions (PanIN, pancreatic intraepithelial neoplasia), which were enhanced in mice fed a high ω-6 fatty acid diet [28]. A nested case-control study that included 731 PC cases showed a significant interaction with leptin levels by follow-up time of more than 10 years. These results showed an association between increasing leptin concentration and PC after a long period of time, which may be explained by the presence of subclinical disease [13].

However, conflicting results on leptin's ability to induce PC proliferation were earlier reported. Intriguingly, a study reported that leptin inhibited the proliferation of MiaPaCa-2 and Panc-1 cells [29]. In line with these findings, it was reported that leptin had no effect on proliferation of Panc-1 and AsPC-1 cells, but enhanced their migration and invasion potentials [11]. In contrast, recent studies showed that leptin induced proliferation in murine Pan02 and human Panc-1 PC cell lines expressing functional OB-R [12], which suggest the effects of leptin could be cell dependent. Leptin also promoted tumor growth and lymph node metastasis in a subcutaneous and orthotopic PC xenograft model [11]. In addition, leptin-inducedactivation of JAK2/STAT3 pathway increased the expression of metalloproteinase-13 (MMP-13) that was positively correlated with OB-R expression in human PC tissues [11]. We previously reported that leptin induces the expression and activation of Notch in breast cancer cells [18]. However, no data on leptin effects on Notch and PCSC have been previously reported in PC.

Notch pathway components are highly expressed in PC [30]. Notch is critical for PCSC maintenance [31], which suggest the activated pathway plays an important role in PC development. PCSC express one or multiple markers and are linked to increased tumorigenicity. PCSC co-expressing CD44/CD24/ESA showed tumor developing potential 100-fold higher than PC that do not express these molecules [32].

The expression of specific Notch molecules has been associated with poor PC prognosis. Notch3 and Hey-1 levels were associated with reduced overall and disease-free survival [33]. Deletion of Notch2 delayed PC development [16, 34]. However, contradictory data have been reported on the oncogenic role of Notch1 in PC. Leptin regulation of Notch in PC may be related to Ras mutations. The coactivation of Kras and Notch1-ICD in mature acinar cells led to a significantly higher number of PanIN lesions compared to activation of Kras alone [35]. In contrast, deletion of both Notch1 alleles in Kras+ mice accelerated PanIN progression, and slightly decreased survival [16, 34]. High expression of survivin (a Notch targeted anti-apoptotic molecule) is associated with shorter overall survival and progression-free survival in patients with resected PC [36]. Present investigations show that leptin induced the expression of survivin in BxPC-3 (wild type Ras phenotype) and MiaPaCa-2 cells (mutated Kras phenotype).

PCSC are regulated by embryonic stem cell (ESC) transcription factors that are aberrantly expressed in PC (SOX2, Oct-4 and NANOG) [37–39]. Oct-4 is an independent factor of poor prognosis that was expressed in 69% of PC and correlated to clinical stage [40]. PC chemoresistance correlated to increased PCSC expressing CXCR4+, CD133+, ABCB1 multiresistance protein and DLL4 [41, 42]. Present data show that leptin induced Notch and PCSC in PC. Additionally, leptin significantly induced Oct-4 expression in PC tumorspheres and xenografts, which were abrogated by IONP-LPrA2. Moreover, analysis of PC xenografts from mice treated with leptin antagonist showed reduced levels of ABCB1+ and CD133+ PCSC and DLL4 in PC cells, which suggests that leptin signaling is important for the expression of these molecules *in vivo*. Therefore, a pivotal mechanism by which leptin increases PCSC populations involves Notch signaling. However, present data show that Notch inhibition via DAPT only partially affected leptin-induction of PCSC. Moreover, PC cell lines showed differential response to DAPT in regards to PCSC. These data may imply that leptin stimulates PCSC expansion by other mechanisms in addition of Notch signaling.

In conclusion, present data strongly suggest that leptin is an important factor involved in PC proliferation and tumor growth, expansion of PCSC populations, which are related to leptin-induced upstream activation of Notch pathway. Furthermore, present data suggest that leptin could contribute to the development of PC through mechanisms involving leptin-Notch axis that increase proliferation, tumorigenesis and PCSC. Additionally, leptin induction of PCSC further suggests that leptin could be involved in the development of PC chemoresistance. In PC mouse models, obesity promoted desmoplasia, tumor growth and chemoresistance [8]. Currently, in contrast with other types of cancer, targeted therapies for PC failed to show relevant activity either alone or in combination with chemotherapy [43]. Thus, leptin signaling could be an important factor for PC development, treatment and chemoresistance. PC treatment using combinations of inhibitors of Notch signaling and Gemcitabine chemotherapeutic drug significantly reduced PC growth [44]. Therefore, targeting leptin-Notch crosstalk could be a potential novel strategy for PC therapy, especially in obese PC patients.

## MATERIALS AND METHODS

### Materials

Recombinant human leptin was purchased from R&D Systems (Minneapolis, MN). Polyclonal antibodies to Notch4, OB-R, and positive controls for these antibodies were obtained from Santa Cruz Biotechnology (Santa Cruz, CA). Notch1 monoclonal antibody was from Sigma (St. Louis, MO). Notch2, Notch3, JAG2 and DLL4 polyclonal antibodies and their positive control were purchased from Abcam (Cambridge, MA). Hey2 polyclonal antibody was obtained from Millipore (Billerica, MA). Survivin polyclonal antibody was from Cell Signaling (Danvers, MA). ESA-FITC monoclonal antibody was purchased from Stem Cell Technologies (Vancouver, BC, Canada). CD24-PE, CD44-APC and Oct-4-PE monoclonal antibodies were obtained from Biolegend (San Diego, CA). CD133-PE monoclonal antibody was purchased from Miltenyi Biotech (San Diego, CA). ABCB1-FITC and H2K-PECy7 monoclonal antibodies were obtained from eBioscience (San Diego, CA). Polyclonal anti-mouse and anti-rabbit conjugated with horseradish peroxidase (HRP) and PAGE-gels were from Bio-Rad laboratories (Hercules, CA). Enhanced chemiluminescence (ECL)-WB stripping buffer and protease and phosphatase inhibitor cocktail were from Thermo Fisher Scientific (Rockford, IL). Fetal bovine serum (FBS) was purchased from Atlanta Biologicals (Norcross, GA), and penicillin-streptomycin cocktails were from Gibco (Grand Island, NY). Dubelco's Modified Eagle's Medium (DMEM) was from American Type Culture Collection (ATCC, Manassas, VA). ALDEFLUOR kit and Mammosphere complete medium were from Stem Cell Technologies (Vancouver, BC, Canada). Vybrant MTT proliferation kit was from Life Technologies (Grand Island, NY). Human leptin ELISA kit was purchased from R&D Systems (Minneapolis, MN). Collagenase IV was purchased from Worthington Biochemical Corporation (Lakewood, NJ). Notch1 monoclonal antibody and its positive control, RIPA Buffer, N-[N-(3, 5-difluorophenacetyl)-L-alanyl]-S-phenylglycine t-butyl ester (DAPT), dimethyl sulfoxide (DMSO) and other chemicals were obtained from Sigma (St. Louis, MO). Leptin peptide receptor antagonist 2 (LPrA2) was synthetized and purified as previously described [45]. This compound binds specifically to OB-R. To increases its effectiveness, LPrA2 was bound to iron oxide nanoparticles (IONP-LPrA2) (Ocean Nanotech, San Diego, CA). IONP-LPrA2 conjugate was analyzed by WB using anti-LPrA2 specific antibodies. IONP-LPrA2 was analyzed for iron content as an estimate of IONP concentration [46].

### Cell culture

Pancreatic cancer cell lines BxPC-3, MiaPaCa-2, Panc-1 (from primary tumor) and AsPC-1 (from metastasis to peritoneum) were obtained from ATCC and cultured in DMEM supplemented with 10% FBS and 1% Penicillin (100U/ml) /Streptomycin (100μg/ml) (P/S). All cells were maintained at 37^°^C in humidified air with 5% CO2.

### ELISA

Conditioned media from BxPC-3 and Panc-1 attached cells cultured for 24 hours and from secondary tumorspheres cultured for one week were harvested and lyophilized using a Modulyo Freeze Drying System (Thermo Fisher Scientific, Waltham, MA). The resulted powders were resuspended in PBS and used for leptin determination via ELISA. DMEM and Mammocult medium were used as controls, respectively.

### Cell cycle assay

For cell cycle assay, Panc-1, MiaPaCa-2, BxPC-3 and AsPC-1 cells were cultured in 6 wells plates until they reached semi confluence (70–80%). Then, cells were starved for 24 h by culturing in FBS-free medium. After starvation, cells were treated with leptin (1.2 nM) and IONP-LPrA2 (0.0036 pM) for 24 h (Panc-1 and MiaPaCa-2) or 72 h (BxPC-3). Next, PC cells were fixed with 100% ethanol, washed and incubated with propidium iodide (PI) staining solution for 40 minutes at 37^°^C. Then, the cell cycle was analyzed using a Cellometer Vision CBA system (Nexcelom Biosciences, Lawrence, MA).

### MTT assay

PC cells were cultured in DMEM supplemented with 10% FBS and 1% P/S. After cells reached 70-80% confluence, they were starved for 24 h in FBS-free medium, treated with leptin (1.2 nM), IONP-LPrA2 (0.0036 pM iron content) or DAPT (20 μM). Cell proliferation rate was determined using Vybrant MTT Kit. The samples were analyzed at 540 nm using a microplate reader (Molecular Devices, CA).

### Western blot

BxPC-3, MiaPaCa-2 and Panc-1 cells were cultured for 24 h as described above. PC cell lysates were prepared using RIPA buffer containing protease/phosphatase inhibitors and subjected to western blot analysis (WB). Fifty μg of total protein was loaded on 8–15% SDS-polyacrylamide gels. Protein bands were transferred to nitrocellulose membranes. After blocking for 30 minutes in 5% skim milk in TBST buffer (TBS plus 0.1% Tween 20), the membranes were incubated with primary antibodies overnight at 4^°^C, followed by incubation with horseradish peroxidase (HRP)-conjugated secondary antibodies and chemiluminescent substrate. Specific antigen expressions were evaluated by capture on X-ray film or using an Image Quant LAS400 system (GE Healthcare, Piscataway, NJ). GAPDH was used as the experimental protein loading control.

### Flow cytometry analysis

PC cells were seeded in 6 wells plates and cultured in monolayer in DMEM supplemented with 10% FBS and 1% P/S until they reached semi confluence (70–80%). Further, PC cells were starved for 24 h (medium with no FBS), then treated with leptin (1.2 nM) and IONP-LPrA2 (0.0036 pM) for variable times (AsPC-1, 24 h; Panc-1, 48 h; BxPC-3 and MiaPaCa-2, 72 h). PC cells were blocked (1% BSA) for 15 minutes at 4^°^C, incubated with monoclonal fluorescent antibodies for detection of cancer stem cells markers for one hour and fixed using 3.7% formalin. For intracellular markers assessment, cells were firstly fixed, then permeabilized for 10 minutes using 0.05% Triton X-100 prior to antibody incubation. PC cells were then analyzed for antigen expression using a flow cytometry Guava system (Millipore, Billerica, MA). To determine the ALDH activity in PC cells, ALDEFLUOR assay was conducted (Stem Cell Technologies, Vancouver, Canada). Briefly, DEAB reagent (inhibitor of ALDH) was added to the control tubes, then ALDEFLUOR reagent was added to all tubes. After 30–60 minutes of incubation at 37^°^C, ALDH+ cells were analyzed for using Guava flow cytometer (Millipore, Billerica, MA).

### Tumorspheres

PC cells were cultured at clonal density (10–30 × 10*^3^* cells/well) in low adherence 6-wells plates containing tumorsphere complete medium supplemented with heparin and hydrocortisone (Stem Cell Technologies). Cells were treated with leptin (1.2 nM), IONP-LPrA2 (0.0036 pM iron content) and DAPT (20 μM). Cells were cultured in humidified atmosphere at 37^°^C and 5% CO2 for 7–10 days. Tumorspheres were defined as spheres with diameter greater than 60 μm and were classified according to their size into three groups: small (60–100 μm), medium (100–200 μm) and large (> 200 μm). To obtain secondary tumorspheres (P2), primary untreated tumorspheres were dissociated with trypsin-EDTA to obtain single cell suspensions which were further treated as described above. Following a similar procedure, P2 tumorspheres were used to obtain P3 tumorspheres. Tumorspheres were counted (total number and based on size) using an optical microscope equipped with a microscope eyepiece reticle (Klarmann Rulings, Inc., Litchfield, NH). Next, tumorspheres were dissociated with trypsin-EDTA and cells were analyzed by flow cytometry as described above.

### PC xenograft mouse model

Protocols for animal studies were reviewed and approved by the Institutional Animal Care and Use Committee of Morehouse School of Medicine. Nude CD1 nu/nu male mice (seven weeks old) were obtained from Charles River Laboratories (Hollister, CA). MiaPaCa-2 tumorspheres were cultured in tumorsphere medium containing 1.2 nM leptin for 10 days. Tumorspheres cultured in regular tumorsphere medium (Stem Cell Technologies) were used as control. Spheres were collected, washed with PBS, trypsinized, washed with PBS again and resuspended at 5,000 cells/ 50 μl saline per injection. The cell suspension was mixed 1:1 with matrigel (Corning, NY) and 100 μl total volume was used per injection. Heterotopic tumors were established by subcutaneous injection of 5 × 10*^3^* MiaPaCa-2 cells derived from tumorspheres into both flanks of each mouse. Twenty one mice were divided into two groups implanted with MiaPaCa-2 cells derived from tumorspheres: 1. Untreated tumorspheres (*n* = 14) and 2. Leptin-treated tumorspheres (*n* = 7). Group 1 was divided into 2 subgroups that received either saline treatment (*n* = 7) or IONP-LPrA2 (0.0036pM; 50μl; i.v.; twice a week) treatment (*n* = 7). The treatment lasted for 7 weeks. Tumor volume was measured using a caliper as previously described [19]. Body weight (BW), food intake and general health condition were recorded weekly. Tumors were harvested and divided into three pieces: one analyzed immediately by flow cytometry, the second was frozen for western blot analysis and the third piece was used for histology (paraffin embedded tissue). For flow cytometry analysis, tumors (0.3 g) were minced in small pieces (2–3 mm), then incubated with 5ml collagenase IV solution (200U/ml) for 2.5 h at 37^°^C. The cell suspension was filtered through a 70 μm-cell strainer to collect tumor cells. Isolation of tumor epithelial cells was performed by repetitive incubation of cells dispersed in culturing medium in 75 cm*^3^* flasks (30 min/37^°^C/5% CO2/3 times). Attached fibroblasts were discarded. The tumor epithelial cells that remained in suspension were used further for flow cytometry analysis as described above. Tumor frozen sections were used to prepare lysates for WB analysis. Fifty μg of total protein was loaded on 8–15% SDS-polyacrylamide gels and specific protein expression was evaluated as described above.

### Histology

Portions of PC xenografts (0.5–1 cm diameter) were fixed overnight in 10% neutral-buffered formalin, then embedded in paraffin, sectioned and stained with hematoxylin-eosin at the Core Pathology Facilities (Emory University, Atlanta, GA). Pictures were made using an Olympus Bx41 microscope.

### Statistical analysis

All experiments and determinations were performed in triplicate. Statistical comparisons were made using student *t* test and ANOVA. Data are presented as means +/– s.e.m. Values for *p* < 0.05 were considered statistically significant.

## SUPPLEMENTARY MATERIALS FIGURE AND TABLES


