# Supplementary material for: Leptin-Notch signaling axis is involved in pancreatic cancer progression
Source: Oncotarget. 2016 Dec 15;8(5):7740–52. doi: 10.18632/oncotarget.13946 (PMC5352357; doi:10.18632/oncotarget.13946)
Supplement: Supplementary file 1 [file oncotarget-08-7740-s001.pdf]

Leptin-Notch signaling axis is involved in pancreatic cancer progression

Supplementary Materials

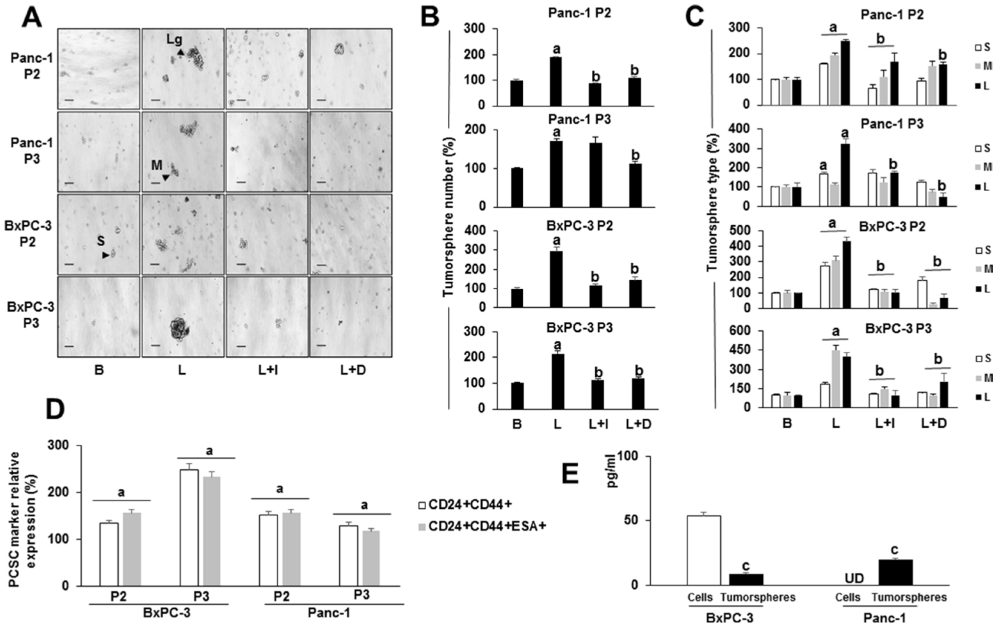

Supplementary Figure S1: Effects of leptin and Notch on secondary and tertiary tumorspheres. (**A**) Representative images of tumorspheres at second (P2) and third passage (P3) (**B**) Number of P2 and P3 tumorspheres. (**C**) Number of P2 and P3 tumorspheres by size (**D**) Relative expression of CD24+CD44+ and CD24+CD44+ESA+ PCSC markers in cells from P2 and P3 tumorspheres (**E**) Leptin determination in conditioned media. BxPC-3 and Panc-1 cells were cultured in basal conditions (medium containing no leptin) for 24 h and for one week to form tumorspheres (P1, P2 and P3) that were treated as described in Materials and Methods. Tumorsphere number and size were determined using a microscope and PCSC markers were analyzed using flow cytometry. Basal condition (untreated) was used as control (100%). Effects of treatment on tumorspheres and PCSC markers were expressed as % of control. Conditioned media was lyophilized, resuspended and used to determine leptin by ELISA. All experiments were performed in triplicate: a: $p \leq 0.05$ compared to control; b: $p \leq 0.05$ when compared to leptin; c: $p \leq 0.05$ when compared to cells. B = basal; L = leptin (1.2 nM); L+I = leptin (1.2 nM) +IONP-LPrA2 (0.0036 pM); L+D = leptin (1.2 nM) + DAPT (20 µM). S = small tumorsphere; M = medium tumorsphere; Lg = large tumorsphere; P2 = secondary passage; P3 = tertiary passage; UD = undetectable.

Supplementary Table S1: Expression of PCSC markers in adherent cells and tumorspheres from PC cell lines

	BxPC-3	Panc-1	MiaPaCa-2
		**%CD24+CD44+**	
**Adherent cells**	**11.6**	**68.3**	**1.2**
**Tumorspheres**	**22.3**	**54.6**	**8.3**
		**%CD24+CD44+ESA+**	
**Adherent cells**	**8.2**	**1.8**	**0.6**
**Tumorspheres**	**18.9**	**2.6**	**0.6**
		**%ALDH+**	
**Adherent cells**	**3.3**	**3.7**	**12**
**Tumorspheres**	**5.8**	**4.25**	**13.69**
		**%CD133**	
**Adherent cells**	**2.5**	**1.8**	**0.54**
**Tumorspheres**	**4.32**	**3.26**	**5.35**

Note: Higher % of cells from tumorspheres express PCSC markers compared to adherent cells grown in basal conditions. PCSC markers were determined by flow cytometry analysis and the experiments were performed in triplicate.

Supplementary Table S2: Histological characteristics of PC xenografts

Treatment	% necrosis	Number of cells in mitosis/10HPF	Average number of cells in mitosis/HPF
Saline	15.6	39	3.9
Leptin-treated tumorspheres	7.2	60	6
IONP-LPrA2	14.7	13	1.3

Note: Leptin increased the number of cells in mitosis in PC xenografts; IONP-LPrA2 treatment decreased leptin's effect. PC xenografts from MiaPaCa-2 tumorspheres pretreated with leptin showed less necrosis. Immunocompromised mice (n = 21) were implanted in their flanks with MiaPaCa-2 cells (5,000 cells/matrigel 1:1) obtained from tumorspheres cultured in basal medium (n = 14) or containing leptin (1.2 nM; n = 7). The mice implanted with PC cells cultured in basal medium were treated with saline (sham control; n = 7) or IONP-LPrA2 (50 µl/0.0036 pM; n = 7) twice per week for 7 weeks. Mice implanted with cells from leptin-treated tumorspheres received saline (n = 7). HPF = high power field.
